# Applicability of Titanium-Based Catalysts in the Photocatalytic Degradation of 2,3,7,8-Tetrachlorodibenzofuran

**DOI:** 10.3390/molecules28227488

**Published:** 2023-11-08

**Authors:** Fatin Samara, Rasha Darra, Ahmed A. Mohamed, Waqas Ahmad, Nedal Abu-Farha, Haesung Lee, Changseok Han, Sofian Kanan

**Affiliations:** 1 Department of Biology, Chemistry and Environmental Sciences, American University of Sharjah, Sharjah 26666, United Arab Emirates; rashadarra@gmail.com (R.D.); nabufarha@aus.edu (N.A.-F.); 2 Department of Chemistry, University of Sharjah, Sharjah 26666, United Arab Emirates; amohamed61@gmail.com; 3 Materials Science and Engineering Program, College of Arts and Sciences, American University of Sharjah, Sharjah 26666, United Arab Emirates; b00092201@aus.edu; 4 Program in Environmental & Polymer Engineering, Graduate School of INHA University, Incheon 22212, Republic of Korea; jellyfish9579@gmail.com (H.L.); hanck@inha.ac.kr (C.H.); 5 Department of Environmental Engineering, INHA University, Incheon 22212, Republic of Korea

**Keywords:** titanium-based catalysts, ELISA, GC/MS, dioxin and furan derivatives, adsorbing material, photodegradation, photocatalysis

## Abstract

Polychlorinated dibenzofurans (PCDFs) are persistent toxic compounds that are ubiquitous in the environment. Nanocomposites of titanium(IV) oxide-vanadium(III) oxide (${\mathrm{Ti}}_{3}V_{2}O_{7}$) and titanium(IV) oxide-silicon dioxide (${\mathrm{Ti}}_{2}{\mathrm{Si}}_{7}O_{30}$) were prepared and spectroscopically analyzed as potential decontaminants for dioxin-like materials. The analysis confirmed a homogeneous morphology with nanoscale particle size. The Ti-Si sample was found to have a high surface area compared to the Ti-V composite. Vanadium(III) oxide (V_2_O_5_) and silicon dioxide (SiO_2_) were chosen as materials for the formation of heterogeneous compounds with titanium(IV) oxide (TiO_2_) because they possess a suitable band alignment with TiO_2,_ thus forming effective photocatalysts. This study evaluated the photodegradation of 2,3,7,8-tetrachlorodibenzo-furan (TCDF) in the presence of Ti-Si and Ti-V oxide composites, which was tested using high- (254 nm) and midenergy (302 nm) UV irradiation sources. While Ti-Si showed success in the photodegradation of 2,3,7,8-TCDF dissolved in a (1:1) methanol–tetrahydrofuran (MeOH-THF) solution, the Ti-V composite proved to be a powerful material in adsorbing TCDF with a high capacity immediately upon mixing. Ti-Si oxide was found to decompose TCDF under the two irradiation sources with 98–99% degradation occurring after 70 min. The use of 254 nm as an irradiation source in the presence of Ti-Si was 4.3 times faster than the analogue reaction irradiated without a catalyst. Byproducts of the degradation were evaluated using gas chromatography–mass spectrometry (GC–MS), resulting in a lower chlorinated congener and less toxicity, as the main degradation product.

## 1. Introduction

Trace contaminants 2,3,7,8-tetrachlorodibenzo-p-dioxin (2,3,7,8-TCDD) and 2,3,7,8-tetrachlorodibenzofuran (2,3,7,8-TCDF) belong to the class of polychlorinated dibenzo-p-dioxins (PCDDs) and polychlorinated dibenzofurans (PCDFs) which are persistent organic pollutants (POPs) of special concern due to their potential toxicity and carcinogenicity [1,2]. Their only natural source in the environment is due to natural disasters such as volcanic eruptions, photolytic reactions of natural substrates, and forest fires, which discharge PCDD/Fs into the environment [3]. PCDD/Fs can be found in soil, dust, and air, and are often present in the environment as byproducts of pollution-related operations associated with sources of combustion, industries, reservoirs, and incineration [4]. Anthropogenic activity leads to the accumulation of dioxins in these sources and includes sewage sludge, municipal waste, waste incineration, petroleum refining, pulp and paper mills, wood burning, and the incomplete combustion of fossil fuels and organic material [5]. 

PCDD/Fs are heavily present in wastewater discharges into surrounding water bodies resulting in the depletion of oxygen and thus, the suffocation of fish, excessive algal growth, and a harmful effect on marine life [6]. Specifically, 2,3,7,8-TCDF tend to bioaccumulate in lipids, and as such can result in health hazards in organisms based on certain characteristics that include but are not limited to exposure time, the life cycle of the organism, and sexuality [7]. To quantify the concentrations of PCDD/Fs in the environment, various techniques have been used: including but not limited to gas chromatography–mass spectrometry (GC–MS), fluorescence spectrometry, ultraviolet–visible (UV–vis) spectroscopy, and enzyme-linked immune sorbent assay (ELISA) toxicology tests [8]. 

The decontamination of environmental matrices is typically performed using techniques such as photocatalysis, photolysis, Fenton-based oxidation, and sonolysis, which have all been successfully implemented and investigated in the literature for the elimination and destruction of PCDDs/Fs [4,9]. In addition, oxidation, combustion, adsorption, and absorption techniques can also be implemented for the decontamination and elimination techniques of PCDDs/Fs [10]. Previous studies have reported on the efficiency of using solid catalysts in the form of coatings, fine powders, or small particles and activating them by a heterogeneous photocatalytic degradation [11]. 

Photodegradation or irradiation is the breakdown of a material upon exposure to a certain UV wavelength [12,13]. Titanium(IV) oxide or titanium dioxide (TiO_2_) is a semiconductor material that has been gaining attention from the scientific community for use as a photocatalyst due its nontoxic nature, strong redox characteristics, and its availability and abundance in nature. TiO_2_ has two major crystallized phases, anatase and rutile, which are highly stable, and another metastable phase, brookite, used as active photocatalysts [14]. The TiO_2_ semiconductor material possesses a large band gap (3.2 eV, anatase), that activates only under UV light [14]. This large band gap allows for a high photochemical stability, which is important for a continuing reaction and reusability in numerous reaction cycles. In addition, when there is a large band gap, the process of photocatalysis occurs by the radiation of light energy that is equal to or greater than the band-gap energy of TiO_2_; this results in the electrons (e^−^) being excited to the conduction band, making holes in the valence band. The photogenerated electrons and holes are active species in photocatalysis for reduction and oxidation (redox) reactions, respectively [14]. TiO_2_’s strong redox ability and above-mentioned properties are what makes it possess desirable photocatalytic properties. 

However, pristine titanium dioxide as a photocatalyst has two main limitations: being inactive in the visible light spectrum and a rapid recombination rate between electron and hole pairs, which results in shortening the life span of the active species for the chemical reactions. To overcome these limitations, band-gap engineering methods have been developed such as defect engineering and heterojunctions [14]. In this paper, TiO_2_ is applied as a heterogeneous catalyst. A heterojunction is when the recombination rate between electron and hole pairs is subdued by the formation of heterogenous compounds between varying crystal phases or materials to elongate the life span of the charge carriers for the chemical reactions. As such, titanium dioxide is widely used as a basis for the formation of a heterogeneous catalyst with distinct properties that include a high surface area, strong metal support interaction, chemical stability, and acid-base properties [15]. Industrial applications of TiO_2_ include water treatment for the photocatalytic degradation of personal care products (PPCPs) and pharmaceuticals [16]. In addition, TiO_2_ is used as a photocatalyst with activated carbon (AC) under ultraviolet light irradiation for degrading hormones and steroids [17]. 

The choice of materials for the titanium-based catalysts in this study are titanium(IV) oxide-vanadium(III) oxide (${\mathrm{Ti}}_{3}V_{2}O_{7}$) and titanium(IV) oxide-silicon dioxide (${\mathrm{Ti}}_{2}{\mathrm{Si}}_{7}O_{30}$), otherwise abbreviated throughout this paper as Ti-V oxide and Ti-Si oxide, respectively. This is due to vanadium(III) oxide (V_2_O_3_) and silicon dioxide (SiO_2_) being semiconductors that have a satisfactory band alignment with TiO_2_ for the formation of heterojunctions or heterogeneous catalysts. Having a suitable band alignment is important for the transfer of photoinduced electrons or holes from one phase or from one semiconductor to another, which results in the separation of electron and hole pairs and leads to a higher chance for reactions to occur. In addition, vanadium in vanadium(III) oxide (V_2_O_3_) is a transition metal, and its use as a metal cocatalyst with TiO_2_ improves the photocatalytic reaction of the semiconductors based on three factors: (1) the activation energy on the surface of the semiconductor is reduced; (2) the electron–hole separation or the separation between the charge carriers is improved in the presence of metal nanoparticles; (3) the occurrence of a plasmonic effect, which is due to surface plasmons being excited, which ultimately improves the harvesting of visible light. Therefore, heterostructured TiO_2_ nanoparticles will possess a higher catalytic activity over pristine ones and thus will have better photocatalytic properties. 

**Scheme 1:**
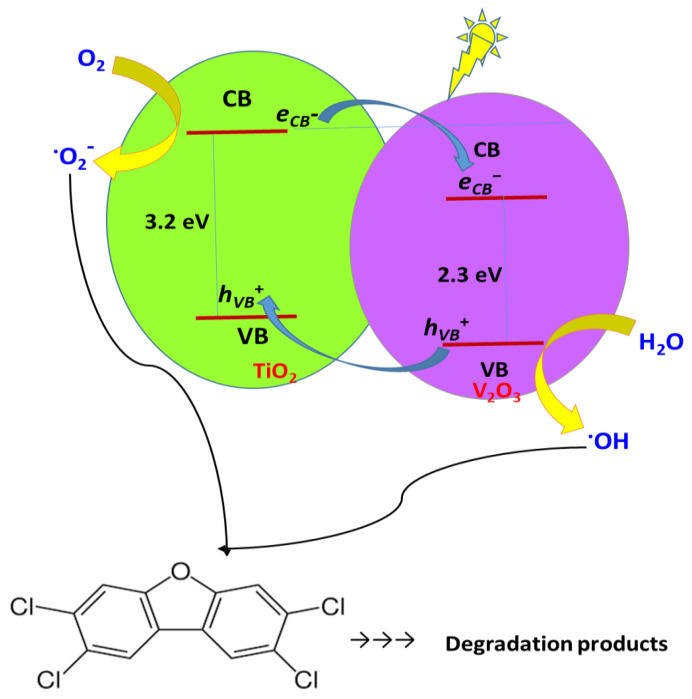
Postulated mechanism for electron transfer in Ti-V oxide composite.

In addition, the surface area of the heterogenous photocatalysts is an important factor to consider but is not the sole desirable characteristic, since the higher the surface area of the photocatalyst, the more contact there is with the solution that is to be degraded. ${\mathrm{Ti}}_{2}{\mathrm{Si}}_{7}O_{30}$ has a high surface area (424 m^2^/g), followed by ${\mathrm{Ti}}_{3}V_{2}O_{7}$ (26.4 m^2^/g). Overall, due to vanadium(III) oxide (V_2_O_3_) and silicon dioxide (SiO_2_) possessing a suitable band alignment with TiO_2_, they have been implemented in photocatalysis reactions as illustrated in Scheme 1 [14,18,19].

To identify the degree of degradation and degradation products, the spectroscopic techniques used are low-temperature solid-state luminescence, Raman and FT-EXAFS, scanning electron microscopy coupled with energy-dispersive X-ray spectroscopy (SEM/EDS), X-ray fluorescence, theoretical calculations, GC–MS, and diffuse reflectance Fourier transform infrared spectroscopy (DRIFT). When it comes to photodegradation, the exact degradation process and degradation products have proven difficult to identify due to the lack of standards, low-resolution peaks that might not match spectral libraries, and differences in mass-to-charge ratios of the identified products [20]. Several photocatalysis applications have successfully been implemented including TiO_2_-SiO_2_ monoliths for the photocatalytic degradation of 4-chlorophenol [11], graphdiyne-doped TiO_2_/SiO_2_ (Ti/Si/GDY) in wastewater treatment [18], and Ti-Si thermal-sprayed coatings to function as photocatalyst coatings on carbon steel and copper substrates [19]. 

In this study, the degradation of 2,3,7,8-tetrachlorodibenzofuran dissolved in a methanol-tetrahydrofuran solution in the presence of two synthesized titanium-based catalysts, titanium(IV) oxide-vanadium(III) oxide (${\mathrm{Ti}}_{3}V_{2}O_{7}$) and silicon-titanium oxide (${\mathrm{Ti}}_{2}{\mathrm{Si}}_{7}O_{30}$) catalysts, using two sources of UV irradiation at 254 and 302 nm, is investigated. Fluorescence spectrometry is used to monitor the photodegradation of 2,3,7,8-TCDD/F by identifying the adsorption capacity of each catalyst. In addition, GC–MS and ELISA toxicology experiments are then used for the characterization and analysis of the degradation byproducts’ relative toxicity.

## 2. Results

### 2.1. Characterization of the Catalysts 

**Figure 1:**
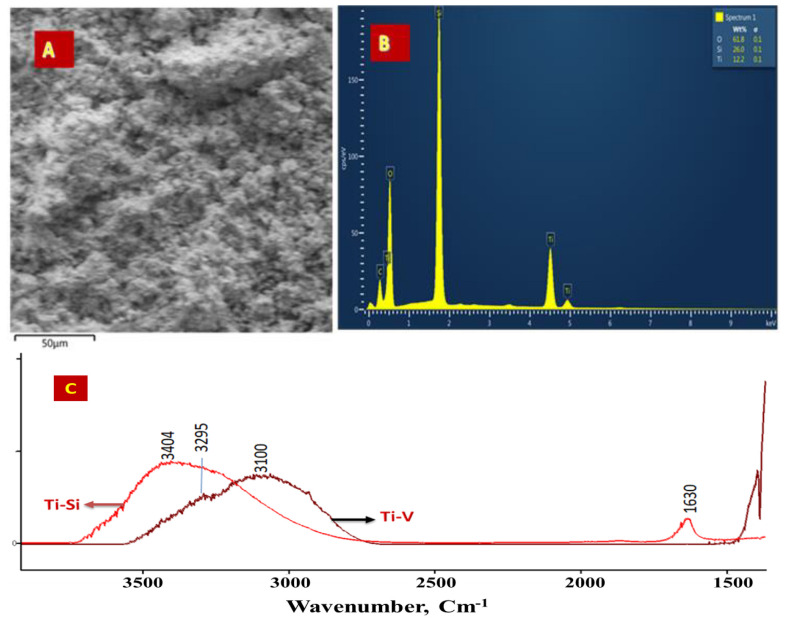
(**A**). SEM image (**B**). EDS analysis of the Ti-Si oxide composite along with (**C**) DRIFT spectra for both materials (not the same *y*-axis scale).

The characterization of the catalysts was performed using various spectroscopic techniques including transmission electron microscope (TEM), SEM/EDS, diffuse reflectance infrared Fourier transform spectroscopy (DRIFT), X-ray diffraction spectroscopy (XRD), and a Brunauer–Emmett–Teller (BET) surface area analysis. Figure 1 shows the SEM and EDS profile for the Ti-Si catalyst (A and B, respectively), and DRIFT spectra for both catalysts (C). SEM-EDS shows a fluffy surface structure for the Ti-Si catalyst, whereby this composite has a similar appearance to silica-based composites made via sol–gel protocols [21,22]. Moreover, the EDS analysis presented in Figure 1B shows a pure Ti mixed silicon oxide composite with an average composition of 12.2% of titanium (Ti), 26.0% of silicon (Si) resulting in a Ti_2_Si_7_O_30_ composition. The data presented in Figure 1B were obtained from a total of four various areas indicating the uniformity of the Ti-Si composite. Likewise, the EDS analysis for the Ti-V catalyst shows a crystalline morphology with 40.7% of titanium and 27.7% vanadium which results in a Ti_3_V_2_O_7_ composition. 

DRIFT spectroscopy is a powerful tool to map the surface properties of various metal oxides. For example, studies have been conducted to identify the adsorption modes of the target molecules, like water, pyridine, and other organic molecules, on the metal oxide surfaces along with mapping the acidic modes that may vary when pyridine binds to Lewis or Bronsted acidic sites, as well as its binding to the adsorbed water moieties through H-bonding. For example, we have previously reported the use of pyridine to fully analyze the surface sites on tungsten(VI) oxide (WO_3_) powder using the pyridine adsorption along with the water desorption from the surface upon evacuation at various temperatures [23,24,25,26]. As seen in Figure 1C, DRIFT spectra for both ${\mathrm{Ti}}_{3}V_{2}O_{7}\;$ and ${\mathrm{Ti}}_{2}{\mathrm{Si}}_{7}O_{30}$ samples show the absence of any C-H or C-C moieties within each composite, indicating the absence of any organic material used during the sol–gel synthetic protocol such as surfactants and precursors [27,28]. This indicates the formation of pure mixed metal oxide composites without any residual carbon-based impurities. The broad peaks that appear above 3000 cm^−1^ represent water adsorbed on the surface which is usually gives an idea about the materials’ surface area before further analysis. The spectra presented are not on the same *y*-axis scale due to the high water adsorption capacity observed for the Ti-Si sample compared to the Ti-V sample.

DRIFT spectra for ${\mathrm{Ti}}_{2}{\mathrm{Si}}_{7}O_{30}$ show broad features in the region 3100–3650 cm^−1^ assigned to the O-H stretching modes of the covalently bonded water to the surface. This broad feature is also associated with the appearance of the sharp bending mode at 1630 cm^−1^. Compared to the recorded DRIFT spectra for the pure TiO_2_ and SiO_2_ powders (see Figure S1), the broad feature seems to be combination of the modes observed on silica and TiO_2_ with a clear shift in the broadening features observed on the TiO_2_ surface as well as the previously reported absorption bands of 3455 cm^−1^ and 1632 cm^−1^ assigned to water-adsorbed moieties [29]. Moreover, the broad features shown for ${\mathrm{Ti}}_{3}V_{2}O_{7}$ that are centered at 3295 cm^−1^ and 3100 cm^−1^ also indicate a shift in the broadening features observed for V_2_O_3_ powders (Figure S1), in which a weak absorption occurs in the range 2700–3100 cm^−1^. Given the strong absorption bands of the Ti-O, V-O, and Si-O modes that appear around 1300 cm^−1^, the region below this wavenumber is opaque and cannot be used to identify any signatures below this range. This problem can be only resolved when nanosized metal oxides can be tailored to apply a thin-film IR absorption band instead of a diffuse reflectance that mainly depends on the particle size and surface area of these oxides [28]. In summary, the DRIFT spectra shown in Figure 1C confirm the absence of any organic contamination on the prepared materials after calcination at 500 °C, as well as the formation of mixed metal oxides with a higher surface area than any of the parent pure metal oxides as presented herein. However, this is not the only possible explanation for the broad peaks, as water has symmetric and antisymmetric stretches that occur in the range of 2700–3700 cm^−1^ and deformation vibrations that occur at around 1650 cm^−1^ [30]. 

**Figure 2:**
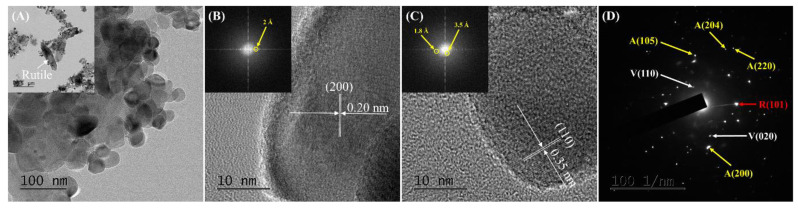
HR-TEM images of Ti-V nanocomposite: (**A**) ×50k and ×10k (inset) images, (**B**,**C**) ×600k image and FFT image (inset), (**D**) SAED patterns (A: anatase, R: rutile, V: V_2_O_5_).

TEM images for the Ti-V oxide catalyst showed a porous, noncrystalline morphology that is rather spread out, with vanadium(III) oxide varying in terms of distribution onto titanium(IV) oxide (Figure 2). The high-resolution TEM (HR-TEM) images in Figure 2 show the shape and size (A), lattice fringes along with the fast Fourier transform (FFT) pattern (B and C), and selected area electron diffraction (SAED) pattern of the synthesized composites. The synthesized Ti-V oxide catalysts were spherical with a uniform size of 41 ± 13 nm as seen in Figure 2A. In the inset image at 10k magnification, interestingly, a crystalline nanorod presumed to be rutile was observed, and nanoparticles were agglomerated around the rutile particle [31,32]. When lattice spacing was analyzed, the measured lattice spacing of 0.20 nm, corresponding to the (200) plane of anatase titanium(IV) oxide, indicated well-crystalized anatase TiO_2_ was produced with the synthesis method. The measured lattice spacing of 0.35 nm, corresponding to the (110) plane that is characteristic of vanadium(III) oxide, confirmed the formation of V_2_O_5_ in the composites with the synthesis method. Also, added fast Fourier transform (FFT) patterns, corresponding to 0.2 nm and 0.35 nm (in Figure 2B and Figure 2C, respectively), corroborated the lattice spacing measurement, indicating the formation of anatase TiO_2_ and V_2_O_5_ in the synthesized composites. Moreover, the SAED pattern in Figure 2D demonstrated the formation of anatase and rutile of TiO_2_ as well as V_2_O_5_ by observing the reflections assigned to (105), (200), (204), and (220) of the anatase, (101) of the rutile, and (020) and (110) of the V_2_O_5_ phase, respectively [33,34,35,36]. According to the results of HR-TEM analysis, it was confirmed that anatase, V_2_O_5_, and rutile were evenly produced with the synthesis method in this study [31]. 

**Figure 3:**
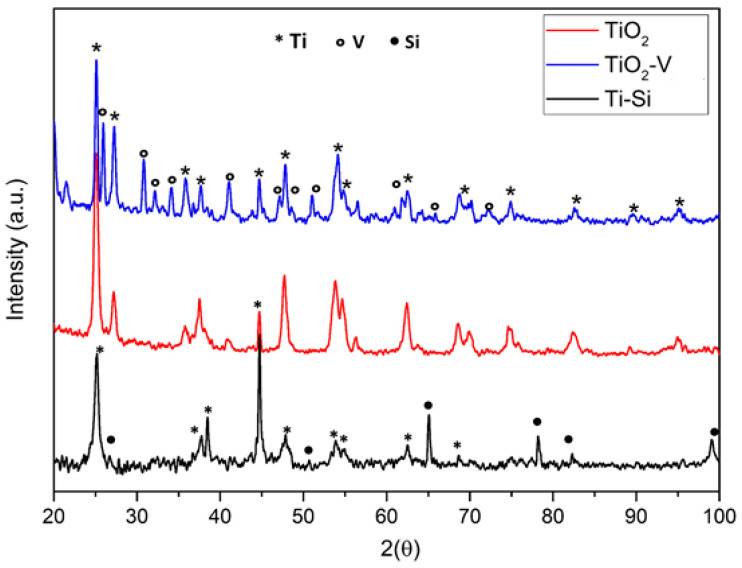
X-ray diffraction pattern of Ti-V and Ti-Si nanocomposites along with TiO_2_ pure sample.

The XRD patterns of all samples are presented in Figure 3. In the two mixed samples, we marked all possible peaks assigned to TiO_2_ along with V_2_O_5_ and SiO_2_ patterns. Both composites were different from the patterns reported for the analogue pure metal oxides. The constituent of V_2_O_5_ in TiO_2_-V_2_O_5_ composite also showed a number of low-intensity broadened diffraction peaks which could be indicative of the nanocomposite formation. The suppression of the distinctive TiO_2_ peak at 2 theta = 25° was one of the obvious characteristics of the TiO_2_-V_2_O_5_ acquired spectrum. These findings demonstrated the creation of nanocomposite materials. However, in the case of TiO_2_-Si, a very sharp peak at 2 theta = 45° made the new phase of TiO_2_ rutile obvious. Additionally, there was a significant suppression of the TiO_2_ peak at 25°. In conclusion, the collected XRD data revealed uniform high-quality nanocomposites. 

**Figure 4:**
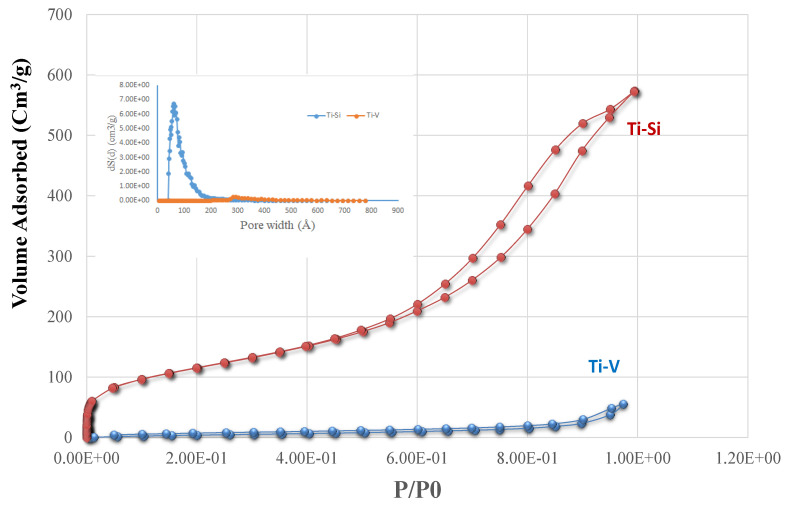
Nitrogen adsorption and desorption isotherms at 77 K of the Ti-Si and Ti-V oxide samples.

Figure 4 shows the nitrogen adsorption and desorption isotherms at 77 K of the Ti-Si and Ti-V oxide samples. Both isotherms show a definite hysteresis at higher P/P_0_ because of the creation of a certain porosity. The calculated surface area for the Ti-Si and Ti-V oxides based on N_2_ adsorption was found to be 424 and 26.4 m^2^/g, respectively. The pore size distribution (the insert in Figure 4) showed that Ti-Si oxide was within the mesoporous scale (pore width range of 40–60 nm) while the Ti-V sample lacked any microporosity compared to the Ti-Si oxide composite.

### 2.2. Photodegradation Experiments

The photodegradation of 10 ppm 2,3,7,8-TCDF in a 1:1 (*v*/*v*) methanol (MeOH)–tetrahydrofuran (THF) mixed solvent was studied in the presence and absence of the Ti-Si oxide and Ti-V oxide catalysts. Titanium-based catalysts were first synthesized, and then, approximately 100 mg of each catalyst was used per sample. The 2,3,7,8-TCDF compound was irradiated using a UV lamp at low- and mid-wavelengths of 254 and 302 nm, respectively. These specific UV wavelengths were selected due to the observed maximum absorption bands of TCDF obtained in the range of 250–320 nm. The photodegradation of 2,3,7,8-TCDF was first monitored as a function of UV irradiation time as monitored using a Varian Cary Eclipse fluorescence spectrometer. Titanium-based oxide samples were then used as heterogeneous catalysts with distinct properties that included a high surface area, strong metal support interaction, chemical stability, and pronounced acid–base properties [15].

**Figure 5:**
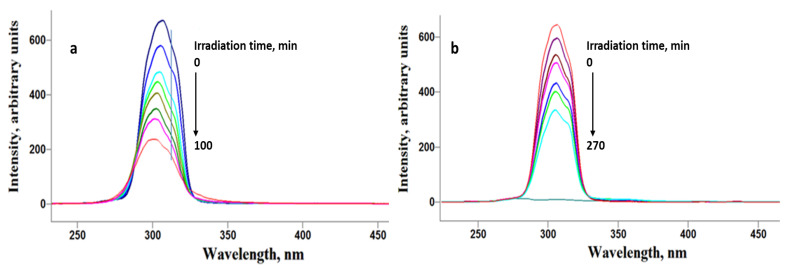
SSLS of the 10 ppm 2,3,7,8-TCDF solution before and after irradiation over 100 min and 270 min irradiation periods using (**a**) 254 nm, irradiation period = 100 min, and (**b**) 302 nm light sources, irradiation period = 270 min, at room temperature.

The batch method was utilized as the kinetic methodology to study the rate of photodegradation of furan, employing a slow irradiation reaction. We have previously reported the luminescence properties of the 2,3,7,8-TCDF [1]. In brief, 2,3,7,8-TCDF showed a strong emission band at 332 nm that corresponds to two absorption (excitation) modes at 250 and 306 nm. The reaction kinetics was monitored using synchronous scan luminescence spectra (SSLS) that were obtained by simultaneously scanning both excitation and emission wavelength monochromators. Herein, we also relied on the profiles obtained with SSLS at Δλ-30 nm for 10 ppm 2,3,7,8-TCDF before and after irradiation at 254 and 302 nm for varying periods of irradiation of 60 min or greater to ensure a clear SSLS graph. In this study, irradiation periods considered were 70, 100, and 270 min. The data presented in Figure 5 show a gradual degradation of the TCDF derivative upon irradiation for 254 nm for 100 min and 302 nm lights for 270 min. The difference in irradiation periods for 254 and 302 nm was not a major factor and simply showed a clearer graph over a period of time greater than 60 min for each SSLS. The data presented in Figure 5 show that TCDF degraded slowly upon irradiation by both UV lights. This is evidenced by the gradual drop in the peak intensity as a function of the irradiation time. Following the 2,3,7,8-TCDF SSLS peak observed at 300–310 nm, a loss of almost 63% was observed upon irradiation at 254 nm whereas, the degradation was up to 45% upon irradiation at 302 nm. 

**Figure 6:**
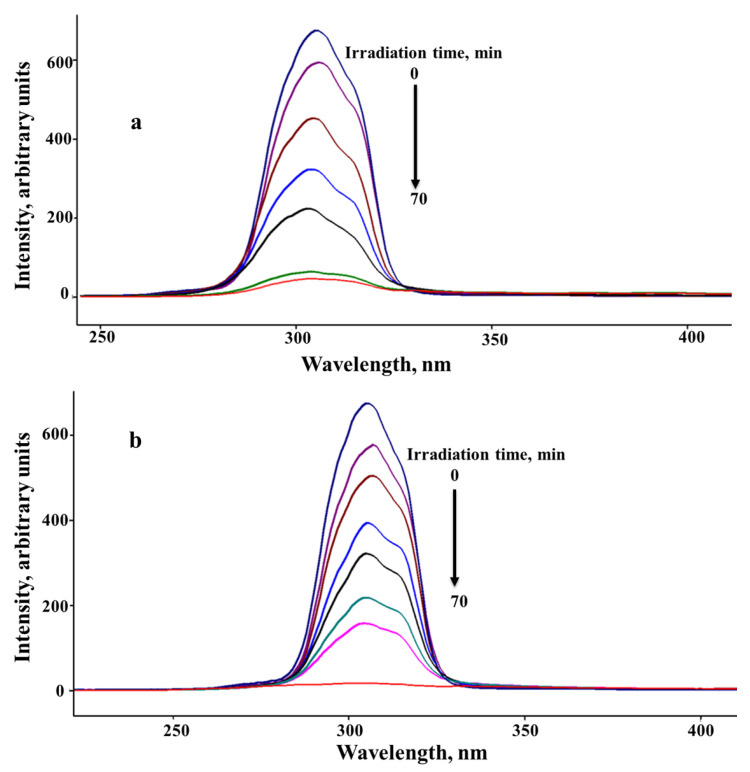
SSLS for 2,3,7,8-TCDF solutions irradiated for 70 min periods in the presence of the Ti-Si oxide catalyst and under (**a**) 254 nm, irradiation time = 70 min, and (**b**) 302 nm UV light sources, irradiation time = 70 min, at room temperature and a catalyst concentration of 100 mg catalyst/7 g furan.

**Figure 7:**
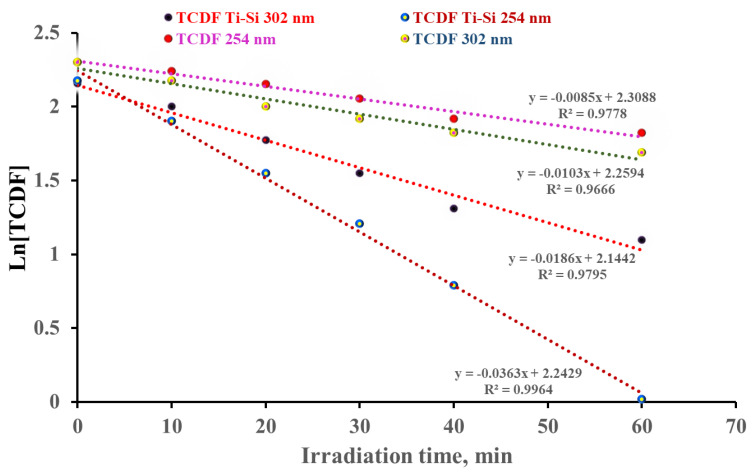
A plot of Ln(TCDF) as a function of irradiation time was obtained for irradiated samples with 254 and 302 nm UV lights in the presence and absence of the Ti-Si oxide catalyst at room temperature and a catalyst concentration of 100 mg catalyst/7 g furan.

To test the catalytic activity of the Ti-Si oxide and Ti-V oxide composites, we conducted a similar protocol in the presence of each catalyst as a solid–liquid heterogeneous catalyst. Interestingly, we observed two different behaviors when the irradiation occurred in the presence of the catalysts. Specifically, the use of a Ti-Si oxide catalyst was found to activate and accelerate the degradation process compared to the uncatalyzed reactions. Figure 6 shows the SSLS of the 2,3,7,8-TCDF sample mixed with 100 mg of the catalysts before and after the irradiation with 254 and 302 nm sources at various times for an irradiation period of 70 min. The irradiation period for the photodegradation experiments must be 60 min or greater to ensure a clear SSLS graph. In this case, a period of 70 min was used. As presented in Figure 6a, the presence of the Ti-Si oxide catalyst enhanced the degradation process to reach 93% and 99% of the initial starting material upon irradiation for 70 min under 254 and 302 nm irradiation sources, respectively. To further analyze the reaction profile, a plot of ln(TCDF) versus irradiation time indicated linear profiles for all irradiation experiments conducted at various irradiation wavelengths in the presence and the absence of the catalyst. A decrease in the TCDF concentration was seen as a function of the irradiated time in the presence and absence of the Ti-Si oxide catalyst. The degradation profile also indicated first-order photodecomposition rates. As shown in Figure 7, the photodecomposition rates of TCDF in the presence of the Ti-Si oxide irradiated with a 254 nm source showed the highest catalytic efficiency for TCDF degradation. The rate constant for the 254 nm irradiated samples in the presence of the catalyst was almost 4.3 times faster than the sample irradiated under similar conditions but without a catalyst. The 302 nm irradiated sample also showed a faster degradation rate than the uncatalyzed sample but with a rate enhancement of 1.8-fold. 

**Figure 8:**
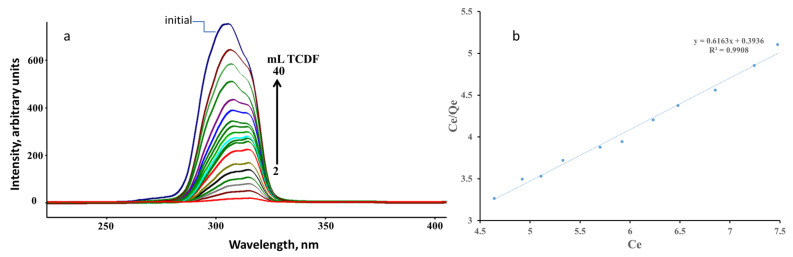
(**a**) SSLS of TCDF over Ti-V oxide monitored after adding various volumes of TCDF and (**b**) the obtained Langmuir isotherm plot of Ce/Qe vs. Ce for TCDF adsorbed on Ti-V oxide.

When comparing both catalysts, we observed that Ti-V oxide showed a high adsorption affinity towards TCDF from the solution, as opposed to Ti-Si. The adsorption of TCDF over the Ti-V catalyst was evaluated by increasing the volume of TCDF added to a 100 mg Ti-V oxide sample as presented in Figure 8a. The figure shows that when small volumes of TCDF solution were exposed to the Ti-V powder, the luminescence profile was quenched. On the other hand, when larger volumes were added, the material started opening until it exceeded the adsorption capacity of the applied material. To evaluate the data collected for Ti-V oxide and study the adsorption capacity of the catalyst, a Langmuir isotherm was constructed based on Equation (1)

C_e_/Q_e_ = 1/(Q_m_ K) + C_e_/Q_m_ (1)

where Q_e_ is the equilibrium concentration of TCDF on the adsorbent, Q_m_ is the monolayer capacity of the adsorbent or the adsorption capacity, K is the adsorption constant, and C_e_ is the equilibrium concentration of TCDF in the solution. A plot of C_e_/Q_e_ versus Ce should be a straight line with a slope of 1/Q_m_ and an intercept of 1/(Q_m_ K) when the adsorption follows the Langmuir equation. The plot presented in Figure 8 shows a good fit for the Langmuir isotherm (R^2^ = 0.9908) with an adsorption capacity of 1.62 mg/g. The adsorption capacity was determined by the slope of the graph 1/Q_m,_ whereby Q_m_ is the adsorption capacity, and was found by calculating 1/0.6163, which resulted in an adsorption capacity of 1.62 mg/g.

### 2.3. Toxicity Comparison of Degraded Sample Using Enzyme-Linked Immunosorbent Assay (ELISA) 

The quality of the degradation products obtained and their variation in toxicity from the photodegradation of TCDF at 254 and 302 nm for catalysts Ti-Si and Ti-V oxides were investigated using ELISA. ELISA functions based on a competitive enzyme immunoassay that relates to the competitive inhibition of a polyclonal antibody that is unique to 2,3,7,8 tetrachlorodibenzo-p-furan. This is due to ELISA having a strong specificity towards PCDDs/Fs with 3–6 chlorines, possessing the highest cross-reactivity for 2,3,7,8-Cl-substituted congeners [37,38]. The concentration and cross-reactivity of the analyte (2,3,7,8-TCDD) affects the binding of the antibody in the assay and it is used as the comparison. Results from the ELISA experiments were expressed in absorbance values and compared to 10 ppm 2,3,7,8-TCDF and MeOH-THF solvent. The results for the solvent blank MeOH-THF with 0 ppm TCDF had an absorbance value of 1.35 A in comparison to the 10 ppm TCDF solution, which reported an absorbance value average of 0.408 A. Thus, the absorbance for the irradiated samples was expected to range (x) from: 0.408 A < x < 1.35 A

**Figure 9:**
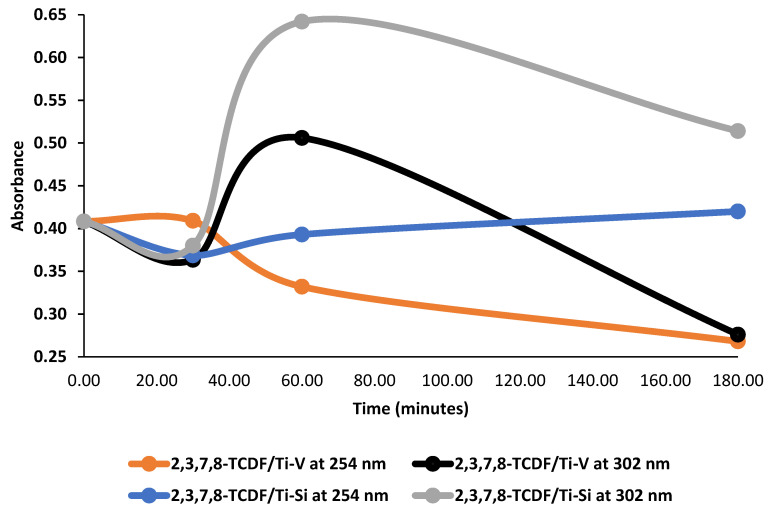
ELISA results for absorbance (A) vs. time (min) for 2,3,7,8-TCDF in the presence of Ti-V oxide and Ti-Si oxide catalysts for irradiation with 254 and 302 nm UV light sources from time zero min to infinity (180 min).

Figure 9 shows the ELISA results for absorbance (A) vs. time (min) for 2,3,7,8-TCDF in the presence of Ti-V oxide and Ti-Si oxide catalysts for irradiation with 254 and 302 nm UV lights from 0 to 180 min. The irradiation of TCDF in the presence of titanium-based catalysts at 302 nm provided a less toxic degradation product at time infinity than TCDF irradiated at 254 nm. At 302 nm, Ti-Si exhibited a lower relative toxicity of final degradation products than Ti-V at time 180 min. Interestingly, at 30 min, all catalysts seemed to produce a more toxic product except for Ti-V at 254 nm, but eventually, this changed after 60 min, when products increased in toxicity. 

The results presented here support the observations presented in Figure 6, where 2,3,7,8-TCDF solutions irradiated for periods of time in the presence of the Ti-Si oxide catalyst under 254 nm and 302 nm UV light sources showed a complete degradation after 180 min, which was similarly observed in terms of the decrease in toxicity at that time (Figure 9). To understand the possible formation of degradation products the case of 2,3,7,8-TCDF was used and is shown in Section 2.4. 

### 2.4. Gas Chromatography/Mass Spectrometry (GC/MS) Quantification and Identification of Degradation Products 

**Figure 10:**
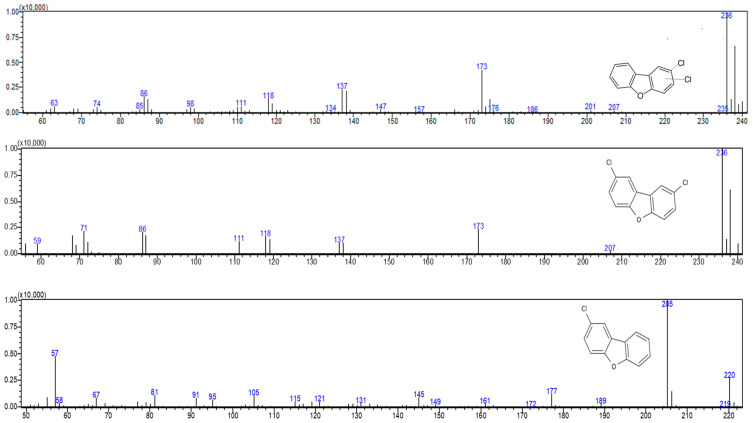
Mass spectra depicted for the identified products for less chlorinated products obtained from TCDF irradiated with a 302 nm UV light for 60 min in the presence of the Ti-V oxide catalyst.

The degradation products of 2,3,7,8-TCDF irradiated at 254 nm and 302 nm, in the absence and presence of catalysts Ti-Si and Ti-V, were examined using GC/MS. Before irradiation, a peak was observed in the GC/MS at 18.48 min. Figure 10 shows the mass spectra of the less chlorinated dibenzofuran obtained from irradiated TCDF samples for 60 min using 302 nm with the Ti-V oxide catalyst. The results showed that with an irradiation in the presence of the Ti-V composite, at all irradiation times, the 2,3,7,8-TCDF peak became invisible at 30, 60, and 180 min of irradiation. In the case of the Ti-Si catalyst, a similar pattern was observed where the 2,3,7,8-TCDF peak became invisible at 30, 60 and 180 min of irradiation, giving rise to the formation of new products. Among the products identified, 2,8-dichlorodibenzofuran (DCDF) at 13.73 min was particularly observed upon irradiation in the presence of Ti-Si at 254 nm, at 30 min, but disappeared at 60 and 180 min, matching the ELISA results for the decrease in toxicity at 60 and 180 min. In the case of an irradiation of Ti-Si at 302 nm, the DCDF appeared at 30 min, disappeared at 60 min, and reappeared at 180 min, which matched the results obtained by ELISA, where the toxicity decreased at 60 min and increased again slightly at 180 min. Other minor products, as depicted in Figure S2, were also observed aside from DCDF, such as 1,5-heptadiene-3,4-diol, tetrahydrofurfuryl alcohol, tricosanol, pentadecanoic acid, octadecanoic acid, hexacosane, 2-(3-tetrahydrofuranyl)-4-ethyl-cyclohexanone, and butanone (see Figure S2 for selected examples). 

## 3. Discussion

The results of this study showed that 2,3,7,8-TCDF photodegraded better at a high-intensity 302 nm UV source, whereby an increase in intensity was observed at a higher rate than with the irradiation of samples with the 254 nm UV source. Results showed that 2,3,7,8-TCDF photodegraded completely in the presence of Ti-Si with a faster rate observed upon irradiation with a 254 nm UV source. In addition, the photodegradation of the TCDF compound without a catalyst indicated 2,3,7,8-TCDF was light-sensitive. In the presence of Ti-Si oxide, 2,3,7,8-TCDF resulted in a better degradation than without the presence of a catalyst. In addition, Ti-Si oxide was the most suitable catalyst relative to all other materials analyzed.

Synthesized titanium-based catalysts were characterized by spectroscopic techniques, confirming a uniform structure, indicating a successful material synthesis with high purity. Titanium dioxide (TiO_2_) was chosen as the base for the preparation of a heterogenous catalyst and a good metal oxide catalyst due to it possessing a plethora of characteristics such as a high surface area, strong metal–support interactions, and acid–base properties [36]. Its high surface area stabilizes the catalyst in its mesoporous structure. This is of crucial significance since the difficulty when it comes to making heterogeneous catalysts often stems from the lack of stability. The active site of catalysts is often blocked during catalysis by the agglomeration of particles, resulting in an unstable catalysis. In addition, at low temperatures and pressures, nanoparticles of titanium oxide possess high activities of reduction and oxidation reactions [39]. 

When comparing the two synthesized catalysts used for the degradation and removal of 2,3,7,8-TCDF from a MeOH-THF solution, ${\mathrm{Ti}}_{2}{\mathrm{Si}}_{7}O_{30\;}$ had a higher surface area (424 m^2^/g), followed by ${\mathrm{Ti}}_{3}V_{2}O_{7}\;$ (26.4 m^2^/g); thus, it had more contact with the 2,3,7,8-TCDF solution. As a result, Ti-Si oxide exhibited the best performance for photocatalytic degradation at the high UV wavelengths of 302 and 254 nm. Similarly, in a study by Robles-Melgarejo et al., TiO_2_-SiO_2_ monoliths were synthesized, whereby the addition of Si contributed to the high surface area of the monoliths that ranged from 528 to 813 m^2^/g, and their effectiveness in being used for the photocatalytic degradation of 4-chlorophenol [11]. On the other hand, Ti-V oxide composites have a higher adsorption capability than Ti-Si oxide composites relative to acid and base sites and the pKa of TCDF. As a result, without the presence of UV radiation, Ti-V oxide proved to be an excellent adsorbing material and the most suitable decontaminating agent. 

Moreover, titanium(IV) oxide-vanadium(III) oxide (${\mathrm{Ti}}_{3}V_{2}O_{7}$) heterogenous catalysts were successfully used in several studies for the destruction and removal of vapor phase PCDD/Fs from the environment. A study by Yu et al. used two types of titanium, nano-TiO_2_ and conventional TiO_2_, as catalyst supports for the preparation of TiV (TiO_2_/VO_x_) catalysts [40]. Results showed that the catalyst activity for nano-TiO_2_-V_2_O_5_ was magnified at an operating temperature range rising from 160 to 300 °C and decreased with a temperature rise to 350 °C [40]. The catalyst activity of Ti-V exhibited such trends due to both PCDD/Fs’ high volatility, whereby the high volatility of organic compounds inhibited the adsorption of PCDD/Fs on the surface of catalysts and suppressed the catalysis reaction that must occur between the catalyst and PCDD/Fs. 

Furthermore, other studies have also proposed the use of Ti-V in the presence of ozone for the catalytic degradation of PCDD/Fs, concluding that the use of an ozone-enhanced oxidation of PCDD/Fs over a TiO_2_-V_2_O_5_-based catalyst was a low-temperature solution that improved the destruction and removal efficiency by increasing the rate of conversion between V^5+^O_x_ and V^4+^O_x_. A study by Ji et al. reported a 99% toxic dioxin destruction efficiency at a temperature of 220 °C [41]. A study by Tang et al. proposed the use of TiO_2_/VO_x_ for the removal of PCDD/Fs from the environment, using 1,2-dichlorobenzene (1,2-DCBz) as a model for the analysis [42]. Results showed that at low temperatures (<200 °C), TiO_2_/VO_x_ catalysts prepared by the mechanochemical method had a higher removal efficiency of 1,2-DCBz (>85%) and stability (>420 min) than the use of the sol–gel method) [42].

It is significant to note that the photodegradation experiments were conducted in a MeOH-THF solution, since TCDF is not soluble in water. As such, the use of methanol, a water-miscible carrier solvent, could have plausibly interfered with the degradation efficiency. A study by J. Arlos et al. evaluated the influence of methanol as a water-miscible carrier solvent for the photocatalytic degradation with TiO_2_ of pharmaceuticals and personal-care products (PPCPs) [16]. The study concluded that trace quantities of methanol affected photocatalytic degradation due to its scavenging effect, specifically for TiO_2_ nanomaterials with low-reactivity properties. Photocatalysis in the presence of methanol resulted in the scavenging of hydroxyl radicals [16]. Silicon has a low reactivity at room temperature and Ti-Si oxide catalyst was used in the photocatalytic experiments in the presence of methanol, which could have affected the degradation efficiency. However, the methanol solvent was maintained at a low concentration, although it could have a more significant effect when present at elevated concentrations.

The toxicity and quality of the degradation products was analyzed using UV light sources. At 302 nm, Ti-Si exhibited a lower relative toxicity of the final degradation product than Ti-V at time infinity. The toxicity was analyzed relative to the parent 2,3,7,8-TCDF, and the generated species could not be specifically identified. However, plausible generated species include the formation of chlorine ions (Cl^−^ and other oxidant species. A study by Amado-Pina et al. investigated an electrochemical advanced oxidation process used in water and wastewater treatment that included a combination of ozonation and in situ cathodic hydrogen peroxide generation to drive a peroxone reaction [43]. The peroxone reaction is simply the reaction of H_2_O_2_ with ozone (O_3_), which produces hydroxyl radicals in aqueous solutions, similar to the hydroxyl radicals that may form in solution as a result of the use of methanol as a water-miscible solvent and its scavenging effect in this study. The study analyzed the electro-peroxone (EP) process of a 4-chlorophenol and the oxidant species produced, degradation pathway, and phytotoxicity [43]. As such, the chlorine ions formed as a result of the photocatalytic degradation of 2,3,7,8-TCDF can act as precursors for the formation of other oxidant species such as oxychlorine anions (perchlorate, hypochlorite, chlorate), facilitate other oxidation reactions, and increase the toxicity of the solution. 

## 4. Materials and Methods

### 4.1. Reagents, Solvents, and Catalysts Preparation

A standard solid of 2,3,7,8-tetrachlorodibenzofuran (2,3,7,8-TCDF) was purchased from Accustandard (New Haven, CT, USA) and dissolved in a solution of (1:1) methanol–tetrahydrofuran (MeOH-THF). All solvents were HPLC grade purchased from Aldrich Chemical Company (St. Louis, MO, USA). Ti-Si and Ti-V oxides were synthesized by the sol–gel method in Span 60 surfactant. To a solution containing 1.0 mole of TiCl_4_ in 30 mL absolute ethanol under a N_2_ atmosphere, 20 mL of Span 60 surfactant was added under continuous stirring. After 1 h, 1.0 mole of SiCl_4_ was added with continuous stirring. The final solution was then stirred for 48 h and then annealed slowly at 500 °C for 3 h. The same procedure was followed to make a Ti-V oxide sample with VCl_3_ added instead of the SiCl_4_ solution. The Ti-Si oxide composite was characterized using SEM-EDX, DRIFT, XRD, BET surface area, and pore size distribution. In addition, Ti-V oxide was analyzed using TEM, SEM-EDS, DRIFT, BET surface area, and pore size distribution, as well as XRD.

For the TEM analysis, a JEOL JEM 2100 high-resolution transmission electron microscope (HR-TEM, JEOL LTD., Tokyo, Japan) was used to analyze the morphology and composite properties of a titanium oxide-based catalyst (Ti-V) by immobilizing it on a TEM grid (Formvar carbon film FCF-400-Cu, Electron Microscopy Science). The nanocomposites in HR-TEM images were measured for particle size, lattice spacing, and FFT using the ImageJ software from the National Institute of Health, USA (https://imagej.net/ij/index.html accessed on 2 November 2023). DRIFT spectra were recorded on a Perkin-Elmer Spectrum 1 equipped with a Praying Mantis Diffuse Reflection accessory from Harrick scientific. Finally, the BET surface areas for both catalysts were measured using BET Surface Area autosorb Analyzer from Anton Paar. 

### 4.2. Photodegradation Irradiation of 2,3,7,8-TCDD and 2,3,7,8-TCDF Experiments

First, 100 mg of each catalyst was used with a 10-ppm solution of 2,3,7,8-TCDF, in a quartz test tube. Among the two synthesized catalysts, Ti-Si has the highest surface area. The higher the surface area, the more the catalyst is in contact with the 2,3,7,8-TCDF solution. A stock solution of 10 ppm 2,3,7,8-tetrachlorodibenzofuran (2,3,7,8-TCDF) was prepared in a 1:1 (50 mL:50 mL) tetrahydrofuran (THF)/methanol mixture to carry out the experiments. The photodegradation of 2,3,7,8 TCDD/F in the presence and absence of a catalyst was performed under a UV light of low intensity (254 nm) and high intensity (302 nm) for 10 min intervals. The irradiation was carried out using a UV lamp (model UVS-28) from VWR Scientific, Inc., with a relative intensity of 1300 W/c at 3 in. Quartz test tubes with an internal diameter of 12.5 mm, a length of 10 mm, and a 1 mm wall thickness were used to irradiate samples, irradiating only one sample at a time. The 2,3,7,8-TCDD and 2,3,7,8-TCDF prepared sample solutions had an exposure distance of 3.0 in, whereby the maximum output of the lamp was reached at that distance. A sample aliquot was taken at 0, 20, 40, and 60 min, and when time went to infinity (approximately 3 h) for further measurements. 

### 4.3. Fluorescence Spectrometry

A Varian Cary Eclipse fluorescence spectrometer was used to monitor the photodegradation of 2,3,7,8-TCDF and to investigate the adsorption trends and adsorption capacity of each catalyst into the sample. The collected sample aliquot was monitored by fluorescence spectrometry every 10 min, over a total span of at least 60 min or more. Irradiation periods of 70, 100, and 270 min were considered. After every 10 min under the UV light, the TCDF solution with a catalyst was centrifuged and the top layer was analyzed by fluorescence spectrometry for measuring the absorbance. Overall, a combination of UV light and fluorescence spectrometry was performed for blank TCDF, TCDF with Ti-Si, and TCDF with Ti–Si oxides, for two wavelengths, 254 and 302 nm. Cary Eclipse-Scan software (2011) was used to scan the excitation, emission, and synchronous spectra for 10 ppm 2,3,7,8-TCDF solution. Synchronous spectra were obtained by fixing a delta of 30 nm between excitation and emission and scanning λ_em_ for the range 200–600 nm (record maximum λ_em_). Graphs of intensity (a.u.) vs. wavelength (nm) were obtained and then utilized to determine the adsorption capacities of each catalyst at 302 and 254 nm. 

### 4.4. Enzyme-Linked Immunosorbent Assay (ELISA) Experiments

ELISA is a toxicology kit used to measure UV–vis absorbance of TCDF samples and determine their toxicity at different stages of photodegradation. An ELISA kit operates based on the competitive inhibition of a polyclonal antibody that is specific to 2,3,7,8-TCDF, whereby the more structurally similar an analyte is to TCDF, the more likely it will bind to the antibody. The steps to conducting the ELISA toxicology test were as follows: First, irradiated TCDF samples were poured into 2 mL amber glass vials and evaporated under a stream of nitrogen by using a particular keeper solution consisting of 100 ppm Triton X-100 in 80:20 methanol:tetraethylene glycol (PEG), that was already provided in the kit. Standards of the keeper solution were then transferred to antibody-coated tubes each with an aqueous sample diluent. Mixtures were incubated overnight whereby the analyte was captured by an immobilized antibody. Tubes were then washed with 0.01% *v*/*v* Triton X-100 in a water solution, and conjugate of a dioxin-like competitor coupled to the enzyme horseradish peroxidase was added as a competitor to the dioxin for the empty binding sites on the antibody that had not been already occupied by the analyte. The tubes were incubated for 15 min, then washed with water, an enzyme substrate was added to the tubes, and the color was recorded, produced by the presence of the captured horseradish peroxidase–competitor conjugate in direct proportion to the amount captured. The quantity of horseradish peroxidase–competitor conjugate bound was inversely proportional to the logarithm of the TCDF concentration in the previous sample incubation step. Tubes were again incubated for 30 min and then a stop solution was introduced to halt any further color development. Lastly, UV–vis absorbance was measured by a spectrophotometer at 450 nm and results were then studied against a standard 2,3,7,8-TCDF at 0 min.

### 4.5. Gas Chromatography–Mass Spectrometry (GC–MS) Analysis 

Irradiated 2,3,7,8-TCDF samples were analyzed by a QP2010 Ultra GC–MS (Shimadzu, Japan) to identify the products formed at different times. A standard RTX-5 capillary column was connected to the gas chromatography, with dimensions of 0.25 µm thickness × 0.25 mm diameter × 30.00 m length from Restek (Bellefonte, PA, USA). The assessment method included the following: First, 1 µL of sample mixture was injected into the GC-MS. The injector temperature was set at 250 °C, and the initial oven temperature was 100 °C held for 1 min. Then, it was raised to 180 °C at 15 °C/min and held for 3 min, followed by a further rise to 290 °C at a rate of 10 °C/min and a hold time of 11 min. The mass spectrometer detector’s ion source temperature was set at 230 °C, and the interface temperature was set at 290 °C. Helium was used as the carrier gas with a flow rate of 1.0 mL/min. TCDF (*m*/*z*) and the newly formed products based on NIST17S library. 

## 5. Conclusions

To conclude, the evaluation of both titanium(IV) oxide-vanadium(III) oxide (${\mathrm{Ti}}_{3}V_{2}O_{7}$) and titanium(IV) oxide-silicon dioxide (${\mathrm{Ti}}_{2}{\mathrm{Si}}_{7}O_{30}$) as catalysts for the degradation and removal of TCDF from a (1:1 MeOH-THF) solution was evaluated in this paper by the photodegradation of TCDF with and without the presence of a catalyst, as well as in the presence of a catalyst but without photodegradation. Results showed that Ti-Si oxide is the most suitable catalyst for photocatalytic degradation at high UV wavelengths of 302 and 254 nm, for an irradiation period of 70 min. An irradiation period of 60 min or greater was chosen to ensure a clear SSLS graph. Ti-Si oxide resulted in a 98–99% degradation of TCDF under the two irradiation sources for an irradiation time of 70 min. The use of 254 nm as an irradiation source in the presence of Ti-Si was 4.3 times faster than the analogue reaction irradiated without a catalyst. Meanwhile, Ti-V oxide proved to be an excellent adsorbing material and suitable decontaminating agent without the need for UV radiation, with an adsorption capacity of 1.62 mg/g for a catalyst concentration of 100 mg catalyst/7 g furan. Vanadium(III) oxide (V_2_O_5_) and silicon dioxide (SiO_2_) were chosen as materials for the formation of heterogeneous compounds with titanium(IV) oxide (TiO_2_) due to them possessing a suitable band alignment with TiO_2,_ thus forming effective photocatalysts with the property of a strong redox ability. This ensures a large band gap that is activated under UV radiation; thus, the heterogenous compounds have a high photochemical stability that leads to a continuing reaction and reusability in numerous photocatalysis reaction cycles. The surface areas of the catalysts were found to be 424 m^2^/g (Ti-Si), and 26.4 m^2^/g (Ti-V). GC–MS was used to analyze the byproducts of degradation, which showed a lower chlorinated congener and less toxicity, as the main degradation product. Overall, this study proves that titanium-based catalysts are promising catalysts to be implemented for the removal of trace contaminant 2,3,7,8-TCDF from the environment. 

## Data Availability

Data is contained within the article.

## Supplementary Materials

The following supporting information can be downloaded at: https://www.mdpi.com/article/10.3390/molecules28227488/s1, Figure S1. DRIFT spectra recorded for commercial powders of TiO_2_ (P-25), fumed silica, and V_2_O_3_. Figure S2. Mass spectra for selected minor products obtained upon irradiation with UV lights in the presence of the Ti-based oxide catalysts.
