# Supplementary material for: Applicability of Titanium-Based Catalysts in the Photocatalytic Degradation of 2,3,7,8-Tetrachlorodibenzofuran
Source: Molecules. 2023 Nov 8;28(22):7488. doi: 10.3390/molecules28227488 (PMC10673168; doi:10.3390/molecules28227488)
Supplement: Supplementary file 1 [file molecules-28-07488-s001.zip › molecules-2662147-supplementary.pdf]

molecules

Supplementary materials for:

Applicability of Titanium-Based Catalysts in the Photocatalytic Degradation of 2,3,7,8-Tetrachlorodibenzofuran

Fatin Samara [1,*], Rasha Darra [1], Ahmed A. Mohamed [2], Waqas Ahmad [3], Nedal Abu-Farha [1], Haesung Lee [4], Changseok Han [4,5] and Sofian Kanan [1,*]

1 Department of Biology, Chemistry and Environmental Sciences, American University of Sharjah, Sharjah 26666, United Arab Emirates; rashadarra@gmail.com (R.D.); nabufarha@aus.edu (N.A.-F.)
2 Department of Chemistry, University of Sharjah, Sharjah 26666, United Arab Emirates; amohamed61@gmail.com
3 Materials Science and Engineering Program, College of Arts and Sciences, American University of Sharjah, Sharjah 26666, United Arab Emirates; b00092201@aus.edu
4 Program in Environmental & Polymer Engineering, Graduate School of INHA University, Incheon 22212, Republic of Korea; jellyfish9579@gmail.com (H.L.); hanck@inha.ac.kr (C.H.)
5 Department of Environmental Engineering, INHA University, Incheon 22212, Republic of Korea
* Correspondence: fsamara@aus.edu (F.S.); skanan@aus.edu (S.K.)

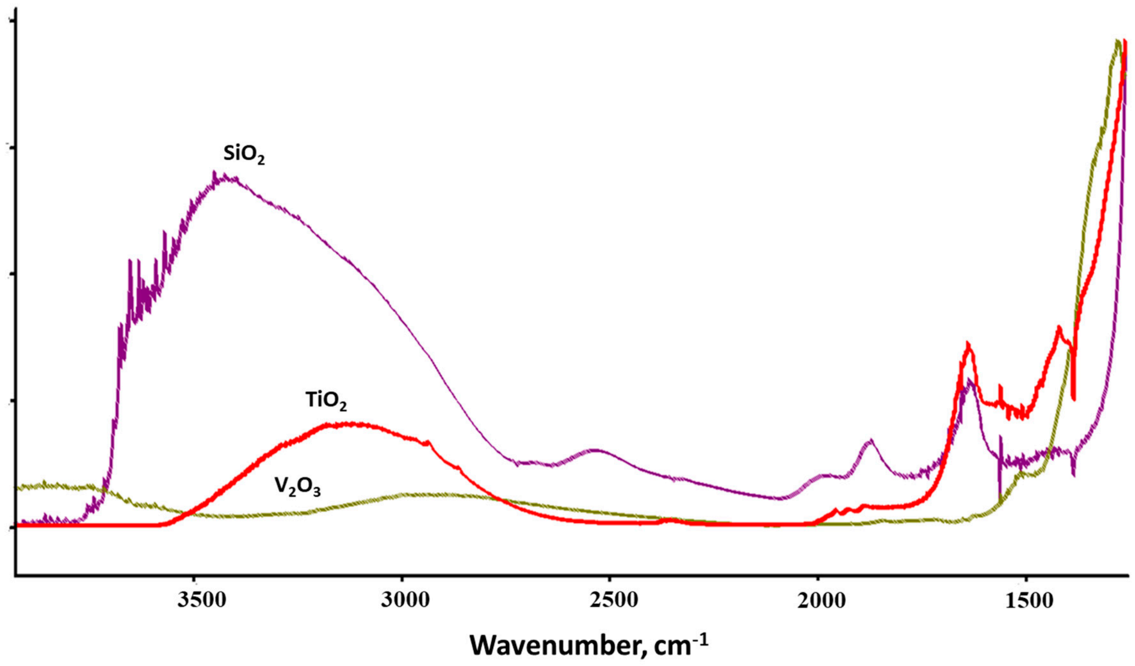

Figure S1. DRIFT spectra recorded for commercial powders of TiO_2 (P-25), fumed silica, and V_2O_3

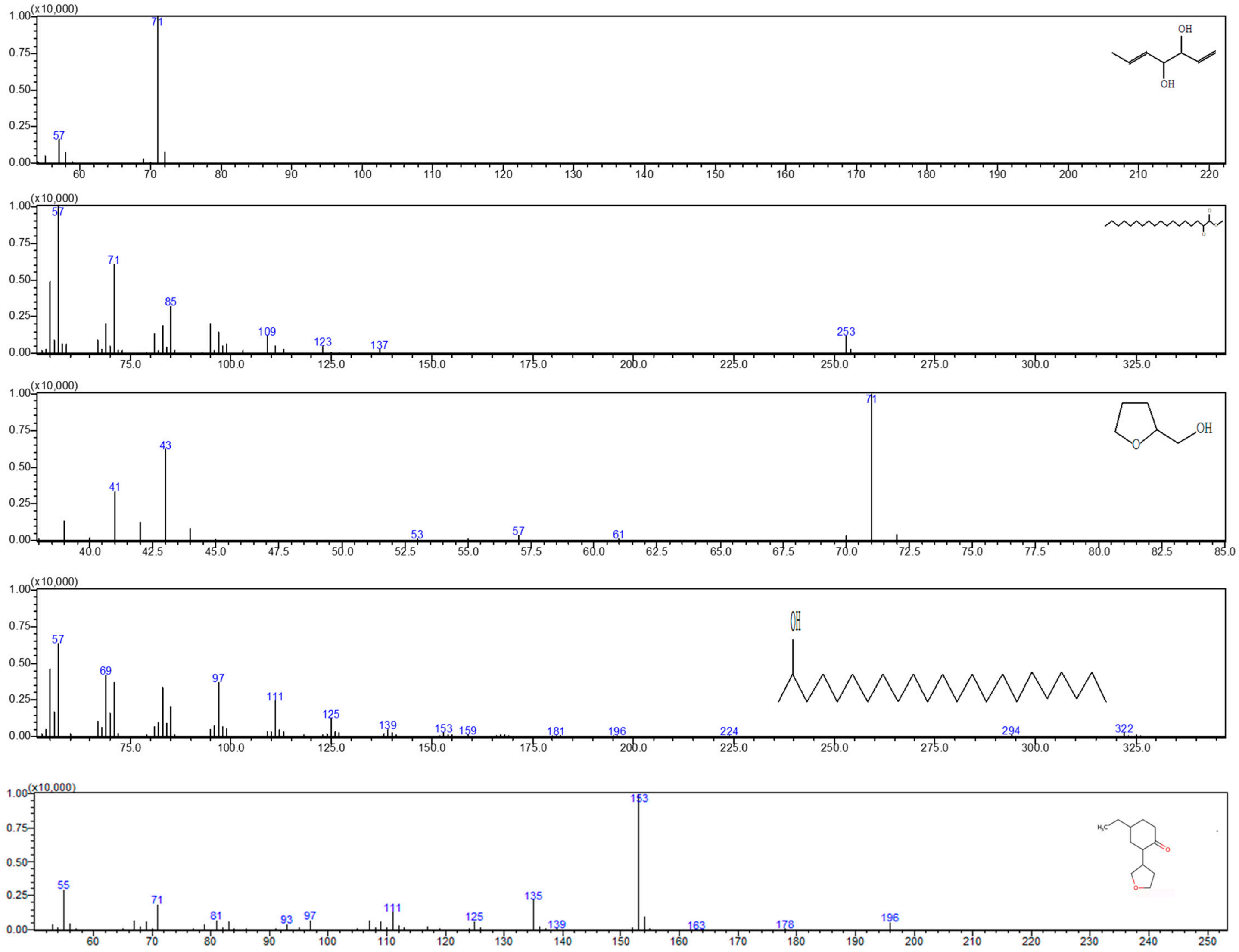

Figure S2. Mass spectra for selected minor products obtained upon irradiation with UV lights in the presence of the Ti- based oxide catalysts.
